# Impact of Peptide Structure on Colonic Stability and Tissue Permeability

**DOI:** 10.3390/pharmaceutics15071956

**Published:** 2023-07-15

**Authors:** Farhan Taherali, Nerisha Chouhan, Fanjin Wang, Sebastien Lavielle, Maryana Baran, Laura E. McCoubrey, Abdul W. Basit, Vipul Yadav

**Affiliations:** 1 Intract Pharma Ltd., London Bioscience Innovation Centre, 2 Royal College Street, London NW1 0NH, UK; f.taherali@sygnaturediscovery.com (F.T.); nerisha.chouhan@intractpharma.com (N.C.); fanjin.wang.20@ucl.ac.uk (F.W.); 2 Sygnature Discovery, Bio City, Pennyfoot Street, Nottingham NG1 1GR, UK; 3 Orbit Discovery, Schrodinger Building, Heatley Rd, Oxford OX4 4GE, UK; sebastien.lavielle@orbitdiscovery.com (S.L.); maryana.baran@npl.co.uk (M.B.); 4 UCL School of Pharmacy, University College London, 29-39 Brunswick Square, London WC1N 1AX, UK; laura.mccoubrey.18@ucl.ac.uk

**Keywords:** oral delivery of biologics and peptides, colonic drug delivery, peptide design and synthesis, gastrointestinal peptide stability, microbiota drug metabolism, bioavailability of biopharmaceuticals

## Abstract

Most marketed peptide drugs are administered parenterally due to their inherent gastrointestinal (GI) instability and poor permeability across the GI epithelium. Several molecular design techniques, such as cyclisation and D-amino acid (D-AA) substitution, have been proposed to improve oral peptide drug bioavailability. However, very few of these techniques have been translated to the clinic. In addition, little is known about how synthetic peptide design may improve stability and permeability in the colon, a key site for the treatment of inflammatory bowel disease and colorectal cancer. In this study, we investigated the impact of various cyclisation modifications and D-AA substitutions on the enzymatic stability and colonic tissue permeability of native oxytocin and 11 oxytocin-based peptides. Results showed that the disulfide bond cyclisation present in native oxytocin provided an improved stability in a human colon model compared to a linear oxytocin derivative. Chloroacetyl cyclisation increased native oxytocin stability in the colonic model at 1.5 h by 30.0%, whereas thioether and N-terminal acetylated cyclisations offered no additional protection at 1.5 h. The site and number of D-AA substitutions were found to be critical for stability, with three D-AAs at Tyr, Ile and Leu, improving native oxytocin stability at 1.5 h in both linear and cyclic structures by 58.2% and 79.1%, respectively. Substitution of three D-AAs into native cyclic oxytocin significantly increased peptide permeability across rat colonic tissue; this may be because D-AA substitution favourably altered the peptide’s secondary structure. This study is the first to show how the strategic design of peptide therapeutics could enable their delivery to the colon via the oral route.

## 1. Introduction

Biopharmaceuticals, also known as biologics, offer an efficacious treatment of numerous chronic and acute diseases due to their ability to selectively bind to pathological targets [1]. However, they must often be delivered parenterally, leading to high treatment costs, an increased systemic exposure, and patient dissatisfaction [2]. Delivery via the oral route could improve many of the drawbacks associated with biologics, although decades of investigation into oral biologic delivery has faced significant challenges [3,4]. The gastrointestinal (GI) tract encompasses a dynamic environment designed for the breakdown of food and protection against microbial pathogens [5]. Gastric and intestinal fluids, host and microbial enzymes, and the epithelial mucous layer can all limit the oral bioavailability of biologics [6,7].

A major challenge faced by peptide-based drugs is their predisposition to enzymatic degradation in the GI tract, resulting in a short local GI and plasma half-lives as well as a reduced therapeutic activity [8,9]. Protease, pancreatic enzymes, endopeptidases and microbial enzymes may rapidly degrade peptides in the GI tract [10]. The endopeptidase trypsin promotes peptide bond cleavage at arginine and lysine amino acids, whilst chymotrypsin causes bond hydrolysis at phenylalanine and tyrosine amino acids. Elastase cleaves peptides bonds in regions high in isoleucine, leucine, valine and serine [10]. Another barrier to the oral delivery of biologics includes the epithelial mucous layer, which lubricates the luminal contents and prevents the epithelial translocation of microorganisms [11]. The viscous mucous often inhibits the permeation of peptides across intestinal tissue and into the systemic circulation [12,13]. If peptides are successful in reaching the epithelial surface, their absorption may still be limited via cleavage by epithelial peptidases, their large molecular size, charge or hydrophilicity [14]. Here, the modification of biologics to enhance passive absorption or active transport across the epithelium could be necessary to facilitate systemic therapeutic activity [4,15,16,17,18].

Oral peptide development has gained increasing attention in the pharmaceutical industry since the approval of oral desmopressin in 1978 [19,20]. This approval represented a breakthrough in biologic delivery; however, the next oral peptide was not approved until 1995 (Neoral^®^, Novartis, Basel, Switzerland). Neoral^®^, a cyclosporine formulation indicated for rheumatoid arthritis, inflammatory bowel disease (IBD) and the prevention of organ transplant rejection, utilises a self-emulsifying drug delivery system in the form of soft gelatine capsules and oral solutions [21,22]. Cyclosporine contains lipophilic moieties, a characteristic cyclic format and N-methylation, which all contribute to its structural stability [23,24]. More recently, in 2019, Rybelsus^®^ (Novo Nordisk, Bagsværd, Denmark) (semaglutide) was approved for the treatment of type 2 diabetes mellitus. Semaglutide is a glucagon-like peptide-1 analogue and is co-formulated in Rybelsus^®^ with the absorption enhancer sodium *N*-[8-(2-hydroxylbenzoyl) aminocaprylate] (SNAC) [25]. Another oral biologic, Mycapssa^®^ (Amryt Pharmaceuticals, Dublin, Ireland), which contains a cyclic peptide, octreotide, co-formulated with sodium caprylate as a permeation enhancer, was approved in 2020 for the long-term treatment of acromegaly [26]. These products employ a combination of chemistry and formulation approaches, facilitating the peptides to maintain their structural stability within the harsh GI environment and achieve absorption across intestinal tissue [1,27,28,29].

Common formulation strategies published in the literature for improving biologic stability and intestinal absorption include nanoparticle or micelle formulation; common molecular design techniques include cyclisation and D-amino acid (D-AA) substitution [30,31,32,33]. Cyclic peptides have been reported to exhibit longer half-lives and better GI stability compared to their linear counterparts [34]. It is also known that the presence of D-AAs within peptides confers greater proteolytic resistance compared to L-amino acids (L-AAs), which are substrates of protease enzymes [35,36].

**Figure 1:**
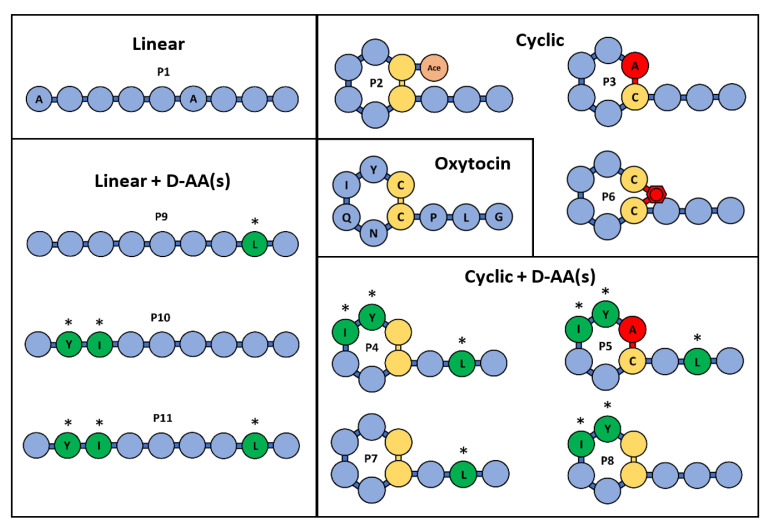
Schematic structures of oxytocin and 11 oxytocin-based peptides. Native oxytocin was used for comparison with oxytocin peptide derivatives. P1 is a linear form of oxytocin, and the P9, P10 and P11 variants are linear forms with varying D-AAs. P2, P3 and P6 are cyclic oxytocin variants (N-acetylated, chloroacetyl and thioether, respectively), and P4, P5, P7, P8 are cyclic forms with varying D-AAs. Amino acids are abbreviated as per: A (alanine); C (cysteine); G (glycine); I (isoleucine); L (leucine); N (asparagine); P (proline); Q (glutamine); Y (tyrosine). The * marker signifies the presence of a D-amino acid.

In this study, we investigated the impact of cyclisation chemistries and D-AA substitution on peptide stability and epithelial permeability in a colonic environment. The colon represents a promising site for oral peptide delivery, due to its lower protease activity and longer transit time compared to the upper GI tract, as well as being an attractive target for the treatment of IBD, colorectal cancer and microbiome perturbations [37,38,39,40,41]. The colonic delivery of gut-restricted peptides for local diseases could facilitate a safer and more efficacious treatment of local diseases, as peptides would be targeted to their site of action with minimal systemic exposure. The treatment of systemic diseases may also be possible for peptides with good colonic permeability; peptides may show better stability in the colon compared to the small intestine due to a lower protease activity. The specific delivery of therapeutics to the colon can be achieved by coating oral solid dosage forms with targeting technologies that resist dissolution in the stomach and small intestine and undergo a pH- and enzymatic-triggered dissolution upon entering the colon [40,42]. Oxytocin was selected as a model peptide as it has an inherently poor GI stability and can be easily chemically modified, allowing several types of cyclisation and D-AA substitution methods to be tested. Eleven oxytocin-based peptides were synthesised for comparison (Figure 1) [42,43]. The peptides were categorised as linear or cyclic, and sub-categorised into two further groups: +/- D-AAs. Peptide stability was assessed using human faecal slurry containing viable microbiota and enzymes from three healthy volunteers. Permeability was evaluated using freshly excised rat colonic tissue, due to the ease of accessibility compared to human tissue. This work is the first to explore how different cyclisation methods and D-AA substitutions could impact peptides’ colonic stability and absorption ex vivo. These chemical strategies could provide valuable insight for the development of oral peptide therapeutics designed to treat both colonic and systemic diseases.

## 2. Materials and Methods

### 2.1. Materials

Chloroacetic acid, α,α′-dibromo-*m*-xylene, ammonia, activated charcoal, trifluoroacetic acid (TFA), piperidine, acetic anhydride (Ac_2_O), 2-(6-Chloro-1H-benzotriazole-1-yl)-1,1,3,3-tetramethylaminiumhexafluorophosphate (HCTU) and diethyl ether (Et_2_O) were obtained from ACROS Organics, UK. Triisopropylsilane (TIS) was obtained from Alfa Aesar, UK. Acetonitrile (ACN) was obtained from Fisher Chemical, UK. Dichloromethane was obtained from ThermoScientific, UK. N,N-Dimethylformamide (DMF) was obtained from Honeywell Riedel-de Haën, Germany. N,N-Diisopropylethylamine was obtained from Pepceuticals, UK. Standard Fmoc-protected amino acids; Fmoc-Ala-OH, Fmoc-Asn(Trt)-OH, Fmoc-Cys(Trt)-OH, Fmoc-Gln(Trt)-OH, Fmoc-Gly-OH, Fmoc-Ile-OH, Fmoc-Leu-OH, Fmoc-Pro-OH and Fmoc-Tyr(tBu)-OH were obtained from Fluorochem, UK. Fmoc-protected D-amino acids; Fmoc-D-Ile-OH, Fmoc-D-Leu-OH and Fmoc-D-Tyr(tBu)-OH were obtained from Novabiochem. Rink amide MBHA resin was obtained from Gyros Protein Technologies, PurePep.

### 2.2. Peptide Synthesis

Peptides were synthesised as C-terminal amides on a Protein Prelude X Peptide Synthesiser at 20-µmol scale (Gyros Protein Technologies, Uppsala, Sweden), using a fivefold excess of Fmoc-amino acids (200 mM) relative to the Fmoc-Rink amide MBHA resin (0.61 mmol/g). Deprotection was performed using 20% piperidine in DMF. Coupling was performed using 1:1:2 amino acid/HCTU/DIEA in DMF. DMF top washes (0.5 min) were performed between deprotection and coupling steps. Amino acids with D-configuration were delivered by the Prelude’s single-shot feature, which presents a zero dead volume.

#### 2.2.1. Cyclisation

The conventional disulfide bond cyclisation was performed in phosphate buffer (10 mM, pH 7.8), the ratio of charcoal to peptide was adjusted to 1:1 (*w*/*w*) and the reaction was stopped after 5 h. Peptides P2, P4, P7 and P8 were engineered to contain the disulfide cyclic structure of native oxytocin.

Thioether cyclisation was used to incorporate a stapling reagent that reacts with two cysteine residues and form a stable covalent linkage to provide a cyclic structure for the P6 oxytocin variant. The proprietary CLIPS^TM^ technology of the biotechnology company, Pepscan, was used to introduce thiol-functional covalent bonds between the cysteines to produce a cross-linking scaffold to induce conformational constraints [44]. The cyclisation procedure was performed using α,α′-dibromo-*m*-xylene in a solution of ammonia in water (pH 8.0) for 1 to 2 h.

Chloroacetyl cyclisation was employed to generate the P3 and P5 oxytocin peptide variants as another type of stable cyclic structure. The N-terminal cysteine was substituted for an alanine, a similar size amino acid, and chloroacetic acid was used as N-terminal capping for the cyclisation strategy. After the deprotection of the cysteine side chain, the spontaneous cyclisation reaction was completed in a few minutes with 20% DIEA in DMF [45].

#### 2.2.2. N-Terminal Acetylation

N-terminal acetylation incorporates an acetyl group at the N-terminus of a peptide [46], producing modifications in protein folding, hydrophobicity and charge [47]. N-terminal acetylation has been implicated in promoting peptide stability, through protecting the NH_2_ terminal end against exopeptidase activity [48,49]. The P2 peptide was engineered to contain an acetyl group, which may confer resistance to proteolysis.

#### 2.2.3. Peptide Purification and Analysis

Peptide cleavage and deprotection were performed with 95:2.5:2.5 TFA/water/TIS for 2 to 3 h. The filtrates were precipitated from cold Et_2_O (50 mL), centrifuged at 5000 RPM (2 × 5 min) and dried. The cleaved products were purified by reverse-phase chromatography using a semi-preparative HPLC, and crude peptides were analysed using an LC/MS 1260 II Series™ HPLC system (Agilent Technologies, Cheadle, UK). Luna C18 Omega Polar Column (150 × 4.6 mm) with parameters: gradient mobile phase 5–60% (ACN with 0.1% TFA), flow rate of 1 mL/min, and UV detection at 214 nm. Mass measurements were carried out on a single quad detector.

### 2.3. Peptide Stability in a Human Colon Model

The human colon model (HCM) was prepared to mimic in vivo colonic conditions and maintain microbial viability [50,51]. Fresh human faecal material was provided voluntarily from 3 healthy individuals who had not taken antibiotics for at least 6 months. Approval for the collection and use of human faecal material was obtained from the National Research Ethics Service (NRES) of Royal Free Hospital Biobank (reference number NC2017.010). Faecal material from the 3 donors was pooled and then diluted within an anaerobic workstation (37 °C, 70% relative humidity) (Elektrotek, Yorkshire, United Kingdom), using an in-house basal growth media to prepare 12.5% or 25% faecal slurries [52]. These slurry concentrations have been used to measure the colonic stability of several drugs and exceed the minimum concentrations required for biorelevant bacterial activity [53]. The samples were homogenised before being sieved (SefarNitex™, pore size 350 µm) to remove fibrous material. Faecal slurry was frozen at −80 °C in aliquots and thawed immediately prior to use.

Peptide stability studies were conducted within the anaerobic workstation (Elektrotek, Yorkshire, United Kingdom). An amount of 2 mg/mL stock solutions of oxytocin and oxytocin variants were prepared and added to HCM at a 1:1 ratio, leading to 1 mg/mL incubation concentrations (*n* = 3 for each peptide). Incubation vials were placed on a 100 RPM shaker and 50 µL samples were withdrawn at specific time intervals (0, 0.5, 1 and 1.5 h) and immediately quenched with 50 µL methanol (1:1) to terminate protease activity. Samples were centrifuged at 16,900 RCF for 10 min at 4 °C, and the supernatant was collected for peptide quantification with a 1260 II Series^™^ HPLC system (Agilent Technologies, Cheadle, UK). A Luna C18 (5 µm, 100 Å, 150 × 4.6 mm) HPLC column was used with a 1.0 mL/min flow rate, an injection volume of 20 µL and mobile phases of 0.1% TFA in water and ACN, with varying gradients for optimised individual peptide quantification to prevent overlap with endogenous components of the HCM.

### 2.4. Peptide Tissue Permeability

Peptide permeability studies were conducted using the NaviCyte Vertical Ussing chamber model (Harvard Apparatus, Cambridge, UK) [54]. Amounts of 250–300 g adult male Wistar rats (*n* = 3) were selected and sacrificed, in accordance with the accepted ethical standards of the National Research Ethics Service (ethical approval number 21/WA/0388). The rat colonic tissues were excised, processed to remove luminal contents and gently washed with Krebs-Ringer Bicarbonate (KBR) buffer (pH 7.4). Each colonic tissue was mounted on an Ussing chamber with the mucosal side facing the apical chamber. The tissue was allowed to acclimatise in the KBR buffer for 20 min. Transepithelial electrical resistance (TEER) across the tissue was measured using an EVOM^TM^ voltameter (World Precision Instruments, Inc., Hertfordshire, UK) and Ag/AgCl electrodes (Harvard Apparatus, Cambridge, UK). TEER values were measured to ensure the tissue integrity was maintained (≥200 Ω cm^2^). The native oxytocin and oxytocin variants at 1 mg/mL concentrations were added to the apical chamber to measure peptide permeation into the tissue and into the basal chamber. Each peptide was tested twice using tissue from the three rats to account for any inter-animal variability. The Ussing chambers were continuously pumped with a supply of carbogen (95% oxygen/5% carbon dioxide) and maintained at 37 °C. An amount of 100 µL samples were taken from the basal side at 1 and 2 h timepoints. At 2 h, the tissues were collected, quenched with 6.25 µL methanol/mg tissue, sectioned with scissors, sonicated for 5 min and centrifuged for 10 min (4 °C, 16,900 RCF), with the supernatant used for peptide quantification. The amount of peptide within the basal chamber and tissue samples was measured with the HPLC method described in Section 3.3 and presented as the percentage of the peptide dose originally applied to the apical chamber. The apparent permeability (*P_app_*) of peptides was calculated using Equation (1):

(1) $$P_{app}=\frac{dQ}{dt\;*\;A\;*\;C_{0}}$$

Equation (1): Apparent permeability (*P_app_*) of peptides across the rat colonic tissue was calculated using the peptide flux (µg/s, *dQ/dt*), the surface area of the tissue (cm^2^) and the initial peptide concentration in the apical chamber (*C*_0_).

### 2.5. Data Analysis and Statistics

All results are presented as mean ± standard deviation and subjected to statistical analysis using GraphPad Prism 9 (GraphPad by Dotmatics). Two-way ANOVA with Dunnett’s multiple comparison test was performed to determine statistical significance between peptides and native oxytocin at multiple timepoints. Results were deemed significant where *p* < 0.05. The physicochemical properties of peptides were estimated using RDKit (version 2021.09.4) in Python (version 3.6.15) via Jupyter Notebook (version 6.3.0) using the peptides’ isomeric SMILES. The Tanimoto similarities of the peptide variants’ molecular structure to native oxytocin was calculated using chemical fingerprints (ECFP6, radius 3, 2048 bits) RDKit (version 2022.9.1) with Python (version 3.7.0) via Google Colaboratory. Chemical similarity maps were generated using RDKit based on bit-vector fingerprints (chirality = true, radius 3, 2048 bits) and Tanimoto similarity [55]. The degradation profiles of peptides were fitted to an exponential decay model in GraphPad Prism 9. A nonlinear regression (one-phase decay, also known as first-order decay) curve was fitted to the peptide stability data with a plateau constraint of 0.0.

## 3. Results and Discussion

### 3.1. Synthesis of Oxytocin-Based Peptides

Table 1 presents the structures and measured molecular weights (MW) of oxytocin and 11 oxytocin-based peptides, alongside their Tanimoto similarities to native oxytocin. Seven cyclic peptides were synthesised; of these, N-terminal acetyl (a known post-translational modification of native oxytocin), chloroacetyl and thioether cyclic bonds were represented, and four peptides also included D-AA substitutions [56]. Four linear peptides were produced, all based on the linear representation of native oxytocin, wherein the two cysteines in oxytocin were replaced by alanines. Of these four linear derivatives, three contained D-AAs, whereby 1, 2, and 3 D-AA insertions were synthesised to allow for the analysis of increasing D-AA insertion on peptide stability and permeability. D-AA substitutions took place at three possible amino acids, tyrosine, isoleucine and leucine, as these were identified as the most protease-susceptible sites in native oxytocin [10,35,57,58]. Native oxytocin (control) was utilised as a comparison against all oxytocin-based peptides.

**Figure 2:**
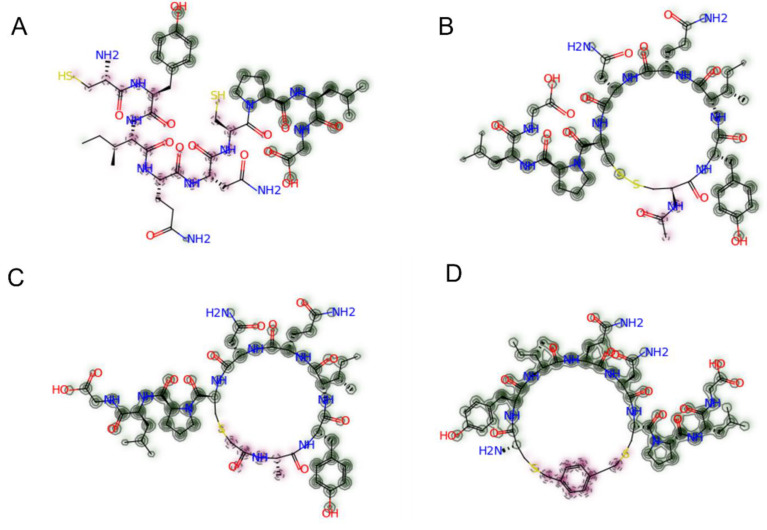
Tanimoto similarity maps for peptide derivatives of native oxytocin. (**A**) The linear derivative P1, (**B**) the N-terminal acetylated cyclisation P2, (**C**) the chloroacetyl cyclisation P3, (**D**) the thioether cyclisation P6. Chemical regions highlighted in green are similar to native oxytocin; regions highlighted in pink are dissimilar to native oxytocin; unhighlighted regions are neutral [55]. Similarity maps were constructed based on bit-vector fingerprints (chirality = true, radius 3, nBits = 2048).

As the synthesised peptides are oxytocin derivatives, their Tanimoto similarities reflect their likeness to native oxytocin. Tanimoto similarity is a chemometric method for the quantification of compounds’ similarity based on their chemical fingerprints [59]. P9, the linear derivative with one D-AA, was the least similar to native oxytocin as demonstrated by a Tanimoto similarity of 0.274. In terms of cyclisation, N-terminal acetylation was found to preserve similarity the most (Tanimoto similarity: 0.836), whereas chloroacetyl cyclisation resulted in the most chemical change (Tanimoto similarity: 0.777). Figure 2 presents the Tanimoto similarity maps for the three non-native cyclisations and the linear derivatives. Here, chemical regions highlighted in green show similarity to native oxytocin, and pink regions show chemical differences [55]. In the variants with the native disulfide cyclisation, substitution with one, two and three D-AAs led to cumulative similarity reductions of 0.148, 0.099 and 0.108 compared to native oxytocin, respectively. As such, both alterations of cyclisation chemistry and D-AA substitution quantitatively reduce chemical similarity to native oxytocin.

The effect of the chemical modifications on peptide affinity for oxytocin binding sites would require further investigation if any of the derivatives were to be further developed. The isoleucine at position 3 of the native oxytocin structure is a requirement for oxytocin receptor binding; therefore, the effect of D-AA substitution is unknown. However, it has been reported that ligands with a close structural similarity to oxytocin are very likely to bind within the polar central pocket of the oxytocin receptor [60]. Further, it has been hypothesised that oxytocin fragments liberated through metabolism may bind to different receptors, thus activating distinct physiological responses [61]. Therefore, modifying the colonic stability of oxytocin derivatives may lead to the preferential activation of certain local and systemic receptors, leading to a targeted physiological modulation.

### 3.2. Insertion of Three D-Amino Acids to Oxytocin Confers Significant Stabilisation

**Figure 3:**
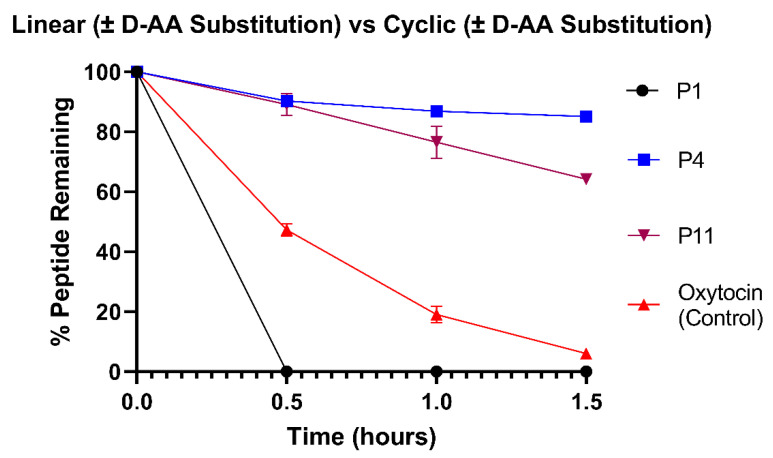
Stability profiles of P1, P4, P11 and commercial oxytocin in the HCM. All variants were compared against commercial oxytocin; *p* < 0.05 at all timepoints from 0.5 to 1.5 h. *N* = 3 for each peptide.

Figure 3 shows the stability of a linear form of oxytocin (P1), an oxytocin derivative with 3 D-AA substitutions (P4) and a linear derivative of oxytocin with 3 D-AAs (P11), compared to native cyclic oxytocin in the HCM. The degradation rate constants and half-lives of all peptides in the HCM are presented in Table S1. Overwhelmingly, P1 was the least stable peptide with total degradation within the first 30 min of incubation. Cyclisation incorporates structural constraints, which reduces access to protease binding and hydrolysis [32,62,63]. The rigidity of the peptides is reflected in the number of rotatable bonds in their structures, with P1 having far more flexibility (29 rotatable bonds) compared to native oxytocin and P4 (17 rotatable bonds). Therefore, the linear P1 peptide was more vulnerable to enzymatic attack than native oxytocin and P4 (*p* < 0.0001), which showed 47.3 ± 1.98% and 90.3 ± 1.57% higher stability at 30 min, respectively. The difference in stability between native oxytocin and P4 revealed that the substitution of 3 D-AAs at the tyrosine, isoleucine and leucine sites significantly improved biostability (*p* < 0.0001). After 1.5 h of incubation, the P4 peptide had retained 85.1 ± 0.21% of its starting concentration, compared to only 6.0 ± 1.36% for commercial oxytocin. The impact of D-AA substitution was further investigated using the linear P11 peptide, comprising three D-AAs. Upon the completion of the 1.5 h study, P11 had retained 64.2 ± 1.18% of its initial concentration. The substitution of L-AAs for D-AAs in peptides can reduce affinity for metabolizing enzymes by altering peptide stereochemistry [35]. Here, we have demonstrated the protective effects of D-AA substitution for oxytocin analogues during incubation in the HCM. These results correspond to studies on other peptides, for example, the effect of D-AA substitution on the resistance of small peptide-based supramolecular hydrogels to proteinase [64]; and elsewhere, the resistance of antimicrobial peptides to intestinal, plasma and bacterial proteases [58].

### 3.3. Chloroacetyl Cyclisation Shows Superior Colonic Stabilisation over Native Oxytocin

**Figure 4:**
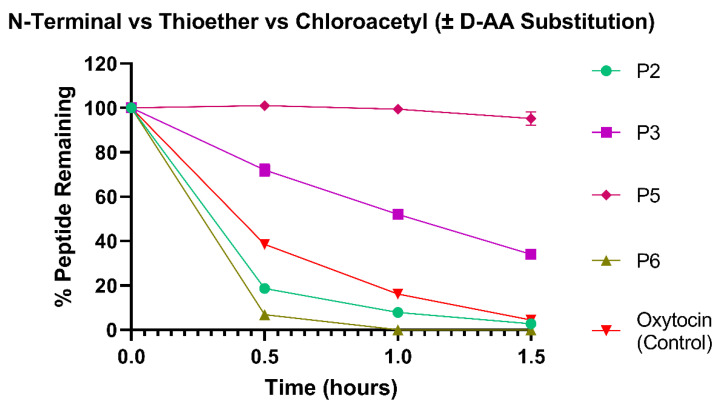
Stability profiles of P2, P3, P5 and P6 variants and commercial oxytocin in HCM. All variants were compared against commercial oxytocin; P3 and P5 were significantly more stable than oxytocin at 1.5 h (*p* < 0.0001). *N* = 3 for each peptide.

Figure 4 shows the stability of oxytocin-based peptides with different cyclisation chemistries in the HCM. Native oxytocin, with its disulfide cyclisation, was compared to N-terminal acetylation cyclisation (P2), chloroacetyl cyclisation (P3), chloroacetyl cyclisation with 3 D-AA substitutions (P5) and thioether cyclisation (P6). The N-terminal acetylation and thioether cyclisations resulted in significantly lower stabilities in the HCM than native oxytocin from 0.5 to 1 h (*p* < 0.0001). After 1.5 h, P2 was degraded by the HCM by 97.2 ± 0.84%, and P6 was 100% degraded in less than 1 h. In comparison, both P3 and P5 with their chloroacetyl cyclisations achieved significantly higher stabilities than oxytocin over 1.5 h (*p* < 0.0001). After 1.5 h incubation, 34.4 ± 0.61% of P3 and 95.2 ± 3.14% of P5 remained intact. These results show that the chloroacetyl ring successfully prevented proteolytic cleavage to a greater extent than the other ring morphologies. The thioether cyclisation present in P6 could have promoted enzymatic cleavage due to its larger size and enhanced flexibility compared to the other cyclisation chemistries tested. The chloroacetyl-cyclised peptides, P3 and P5, showed the highest stabilities, potentially as the chloroacetyl moiety was added to the N-terminal amine of an alanine unit, thus protecting this susceptible site from enzymatic attack. Further research could examine whether the superiority of chloroacetyl cyclisation for peptide stabilisation is specific to oxytocin-like peptides, or if it is a molecular design technique that could be employed to improve the colonic stability of diverse peptides. The almost total stability of P5 in the HCM demonstrates that cyclisation and D-AA substitution resulted in an additive resistance to metabolism. Therefore, the inclusion of multiple stability-enhancing moieties in peptides intended for oral delivery can be deemed as a beneficial molecular design strategy.

### 3.4. Site of D-Amino Acid Substitution May Be more Important Than Number of Substitutions

**Figure 5:**
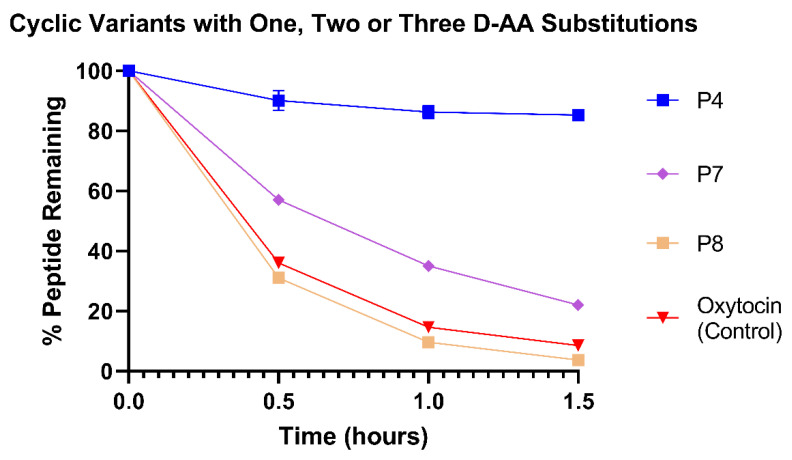
Stability profiles of P4, P7 and P8 variants and commercial oxytocin in HCM. All variants were compared against commercial oxytocin; all peptides had significantly different concentrations compared to oxytocin at all timepoints from 0.5 to 1.5 h. *N* = 3 for each peptide.

To investigate whether the site and number of D-AA substitution influence stability, we compared the stabilities of P7 (1 D-AA: D-Leu), P8 (2 D-AAs: D-Tyr, D-Ile) and P4 (3 D-AAs: D-Tyr, D-Ile, D-Leu) in the HCM (Figure 5). Somewhat unexpectedly, P8 was less stable than P7 (*p* < 0.0001), despite P8 incorporating one D-AA more than P7. At 1.5 h, 22.3 ± 0.85% of P7 remained in the HCM compared to only 3.70 ± 0.43% of P8. This finding suggests that the site of D-AA substitution may be more important for inferring proteolytic stability than the number of D-AA substitutions. Both of P8′s D-AAs were located within its disulfide ring, whereas P7 contained its D-Leu at position 9 of its linear portion following the disulfide ring. Therefore, it can be conferred that the leucine in the linear portion of the amino acid chain was more susceptible to cleavage than the amino acids within the ring, potentially as the ring sterically hinders the binding of metabolising enzymes. That said, the increased stability of P4 compared to P7 validates that the disulphide ring was still susceptible to proteolytic cleavage; substituting D-AAs at the tyrosine and isoleucine sites within the ring as well as the leucine in the linear peptide portion resulted in P4′s high stability (85.3 ± 0.90%) after 1.5 h. Both P4 and P7 were significantly more stable than native oxytocin at all timepoints from 0.5 to 1.5 h (*p* < 0.0001). In comparison, P8 was significantly less stable than native oxytocin from 0.5 to 1.5 h (*p* < 0.05).

### 3.5. Number of D-Amino Acid Substitutions in Linear Analogues Positively Correlates with Colonic Stability

**Figure 6:**
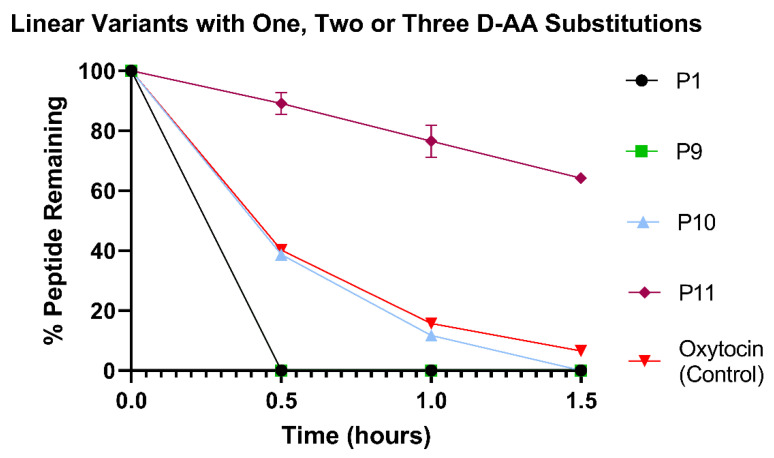
Stability profiles of P1, P9, P10 and P11 variants and commercial oxytocin in HCM. All variants were compared against commercial oxytocin; at 1.5 h P1, P9 and P10 were significantly less stable than oxytocin (*p* = 0.0053) and P11 was significantly more stable (*p* < 0.0001). *N* = 3 for each peptide.

The impact of the number of D-AA substitutions on a linear form of oxytocin was assessed in the HCM (Figure 6). Both P1 (no D-AAs) and P9 (one D-Leu) were rapidly degraded in less than 30 min. This demonstrates that the presence of a single D-AA in the linear form of oxytocin was not sufficient to stabilise the peptide. Linear peptides are inherently more unstable than cyclic analogues, and even the substitution of two D-AAs in P10 did not improve upon the stability of native oxytocin. However, a positive correlation between the number of D-AAs and stability in the HCM was observed, as P10 was more stable than P1 and P9, whilst P11 with three D-AAs was the most stable linear derivative of oxytocin. After 1.5 h of incubation, 64.2 ± 1.18% of P11 remained intact compared to only 6.5 ± 0.0% of native oxytocin, a significant difference (*p* < 0.0001). This proves that D-AA substitution is a viable strategy for increasing peptide stability in the colonic environment and can significantly improve upon native peptide stability even when used without other stabilisation techniques, such as cyclisation.

### 3.6. Number of D-Amino Acid Insertions Positively Correlates with Colonic Tissue Permeability

**Figure 7:**
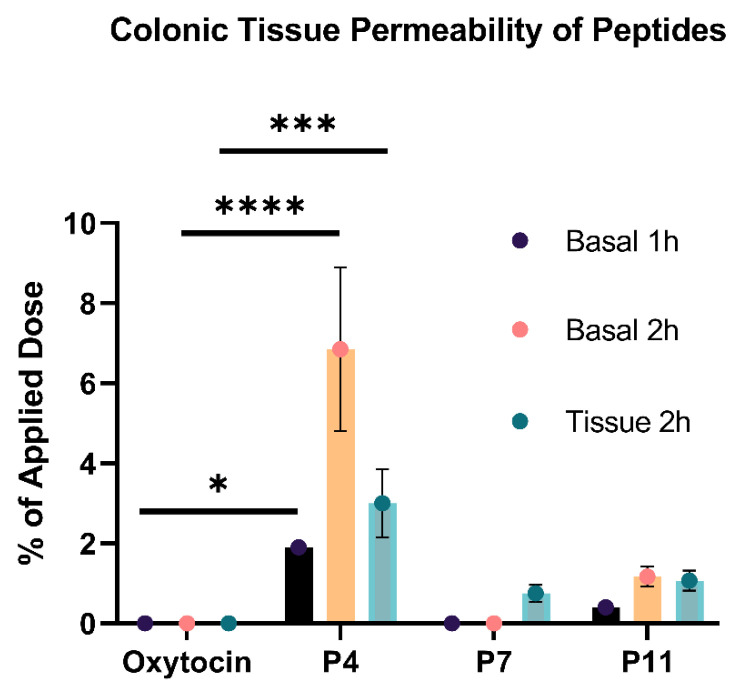
Rat colonic tissue permeability of P4, P7, P11 and commercial oxytocin. All variants were compared against commercial oxytocin; the * symbol: *p* < 0.05; *** symbol: *p* < 0.001; **** symbol: *p* < 0.0001. *N* = 2 for each peptide.

Following investigation in the HCM, three of the most stable peptides, P4, P7 and P11, were selected for the analysis of their permeability across rat colonic tissue. The P_app_ of the peptides at 1 and 2 h is presented in Table S2. Native oxytocin was not detected in tissue or in the basolateral chamber at 1 or 2 h, indicating that it did not permeate colonic tissue at all (P_app_ = 0.00 cm/s). Only P4 (disulfide cyclisation and three D-AAs) had a significantly higher tissue permeability than native oxytocin (Figure 7). At 2 h, 3.0 ± 0.85% of the original dose of P4 was detected within the colonic tissue and 6.85 ± 2.05% was detected in the basolateral chamber, indicating permeation. The P_app_ of P4 at 1 h was 5.70 × 10^−5^ cm/s and at 2 h was 2.06 × 10^−4^ cm/s, signifying good permeability. In comparison, P7 (disulfide cyclisation and one D-AA) and P11 (linear with three D-AAs) did not have a significantly higher permeation than native oxytocin (P_app_ at 2 h: 0.00 and 3.52 × 10^−5^ cm/s, respectively). These results demonstrate that the substitution of three D-AAs in combination with the disulfide cyclisation of native oxytocin was necessary to increase epithelial permeability. As such, this combined approach could be a promising technique for the colonic delivery of peptides intended for local and systemic action. This modification could be beneficial for increasing the colonic permeability of other peptides indicated for IBD or colorectal cancer [65]. Cyclisation can facilitate peptides to adopt open and closed molecular conformations, based on intramolecular hydrogen bonding, that facilitate movement across the cell membrane [66]. It is thought that bioavailable cyclic peptides first anchor to the membrane in their open or closed conformation; they then orient within the membrane, and subsequently adopt their closed conformation to passively diffuse across the lipid bilayer [67]. As D-AA substitution here was necessary to significantly increase tissue permeability, it may be that the altered secondary structure attained by D-AA substitution aided the anchoring, orientation and/or passive diffusion of the cyclic peptide across the membrane [68,69]. As the tissue permeability and retention of P4 is still relatively low, formulation with permeation enhancers could be considered if P4, or similar peptides, were to be further optimised for colonic permeability [70,71].

This paper has revealed several molecular modifications that can improve the stability and permeability of oxytocin in a simulated colonic environment. As with most studies, there are limitations to our methods, namely the comparability of the HCM and permeability model to in vivo human physiology. Firstly, human faecal samples were used to simulate the enzymatic activity of the colon. Faeces are easily obtainable and contain the majority of microbial species found in the colon [72]. Moreover, for colonic drug delivery, the stability of small-molecule drugs in the presence of faecal slurry has been shown to correlate well with in vivo bioavailability [73,74]; yet, the correlation between peptide stability in faecal slurry and in vivo bioavailability has not yet been proven. As such, aspirates of human colonic fluid could provide a more accurate in vitro model, although these can be difficult to obtain. A second limitation of this study was the use of rat colonic tissue in an Ussing chamber to model colonic peptide permeability. The Ussing chamber is an accepted technique for predicting intestinal permeability in preclinical drug development [53]. However, human intestinal tissue (rather than rat) would likely provide a closer approximation of in vivo peptide permeability due to the presence of relevant transporters, enzymes and other anatomical elements [75]. As with colonic fluid, human colonic tissue is difficult to source and may be associated with a particular disease state. Hence, animal tissue provided a close alternative in this study.

A valuable next step for this work would be to investigate the effects of cyclisation and D-AA substitution on a wider range of therapeutic peptides’ stabilities and permeabilities. This would validate whether the modifications can be applied to structurally diverse peptides to improve their bioavailability following colonic delivery. In addition, the effects of cyclisation and D-AA substitution on peptide potency could be examined in more detail. The potency of associated metabolites could also be investigated. Effects on target binding would likely be peptide-specific; thus, binding studies could be completed for the peptides with substantial potential for colonic delivery. Key indications could include IBD, colorectal cancer and microbiome dysbiosis [37,38,39,40]. As most peptide drugs are currently administered parenterally, there is significant opportunity for the development of oral peptide formulations. Clinical advantages of delivering peptide drugs to the colon via the oral route include simple and non-invasive administration, good patient compliance and the direct access to local colonic targets. Following the successful in vitro analysis of peptide stability, permeability and binding affinity, candidates could be progressed to assessment in in vivo models [65].

## 4. Conclusions

In this study, the impact of various cyclisation chemistries and D-AA substitutions on the colonic stability and colonic tissue permeability of oxytocin and 11 oxytocin-based peptides was investigated. Compared to native oxytocin with its disulfide ring, the incorporation of a chloroacetyl ring significantly increased peptide stability in the HCM. Comparatively, N-terminal acetylation and thioether cyclisation decreased stability. The substitution of three D-AAs into the chloroacetyl analogue resulted in over 95% stability after 1.5 h incubation, and three D-AAs in the disulfide variant achieved over 85% stability. Linear forms of oxytocin were inherently less stable in the colonic environment compared to cyclic versions due to their heightened vulnerability to enzymatic cleavage. However, the addition of D-AAs to the linear forms improved stability with three D-AA substitutions, achieving a significantly higher stability than native oxytocin. P4, the cyclic analogue containing three D-AAs, was observed to permeate rat colonic tissue to a greater extent than oxytocin, a linear analogue with three D-AAs, and a cyclic analogue with one D-AA. Here, we have demonstrated that a combination of cyclisation and D-AA substitution chemistries enhanced peptide stability and tissue permeability. These findings promote the use of multiple techniques for improving the oral bioavailability of therapeutic peptides, including peptides intended for colonic delivery. Future work should validate the effectiveness of these strategies for diverse peptide structures in vivo, facilitating the move towards oral peptide drugs for an improved treatment of colonic and systemic disease.

## Figures and Tables

**Table 1:** The properties of oxytocin and 11 synthesised oxytocin-based peptides. Amino acids are abbreviated as per: A (alanine); C (cysteine); G (glycine); I (isoleucine); L (leucine); N (asparagine); P (proline); Q (glutamine); Y (tyrosine).

Peptide	Structure	Amino Acid Sequence	MW (g/mol)	Tanimoto Similarity to Native Oxytocin
**Oxytocin** **(Control)**	Cyclic (disulfide)	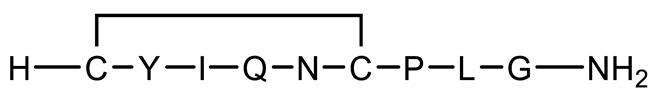	1007.2	1.000
**P1**	Linear	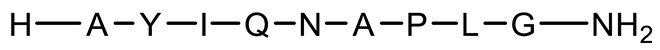	946.1	0.331
**P2**	Cyclic (N-terminal acetylated)	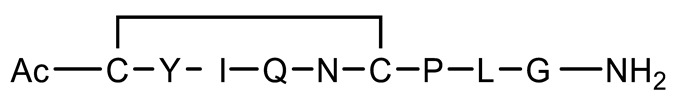	1050.2	0.836
**P3**	Cyclic (chloroacetyl)	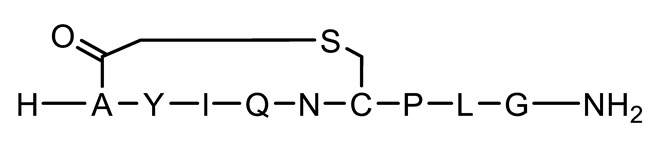	1018.2	0.777
**P4**	Cyclic (disulfide) with 3 D-AAs	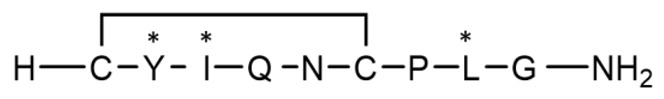	1008.2	0.645
**P5**	Cyclic (chloroacetyl) with 3 D-AAs	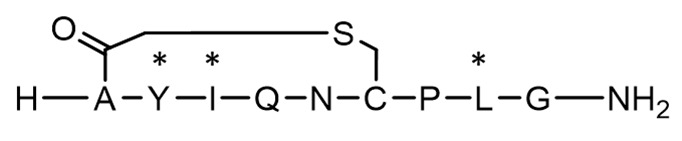	1018.2	0.609
**P6**	Cyclic (thioether)	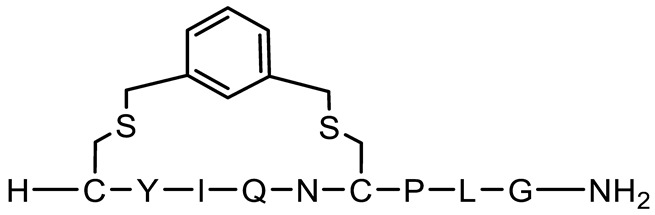	1112.3	0.821
**P7**	Cyclic (disulfide) with 1 D-AA	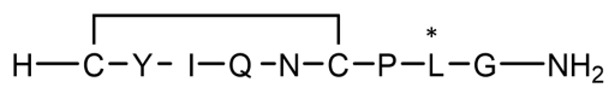	1008.2	0.852
**P8**	Cyclic (disulfide) with 2 D-AAs	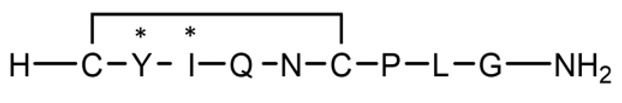	1008.2	0.753
**P9**	Linear with 1 D-AA	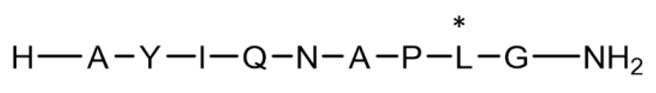	946.1	0.274
**P10**	Linear with 2 D-AAs	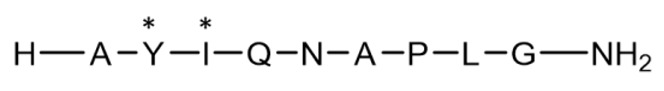	946.1	0.352
**P11**	Linear with 3 D-AAs	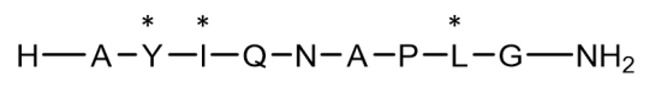	946.1	0.286

* D-Amino acid substitution.

## Data Availability

The data presented in this study are available in the figures and tables. Please direct requests for raw data to the corresponding authors.

## Supplementary Materials

The following supporting information can be downloaded at: https://www.mdpi.com/article/10.3390/pharmaceutics15071956/s1, Table S1: The degradation rate constants and half-lives of peptides in the human colon model; Table S2: The apparent permeabilities of the peptides tested in the Ussing chamber model, using rat colonic tissue.
