# Supplementary material for: Impact of Peptide Structure on Colonic Stability and Tissue Permeability
Source: Pharmaceutics. 2023 Jul 15;15(7):1956. doi: 10.3390/pharmaceutics15071956 (PMC10384666; doi:10.3390/pharmaceutics15071956)
Supplement: Supplementary file 1 [file pharmaceutics-15-01956-s001.zip › pharmaceutics-2482780-supplementary.pdf]

Table S1. The degradation rate constants and half-lives of peptides in the Human Colon Model (HCM) when fitted to an exponential decay model. NC: not computable, as P1 and P9 were entirely depleted between 0.0 - 0.5 hr. *P5 did not fit to the exponential decay model as it was highly stable in the HCM.

Peptide	Goodness of fit to first order degradation (R^2)	Degradation rate constant (% peptide/hour)	Half-life (hours)
Oxytocin	0.9952	1.620	0.4278
P1	NC	NC	NC
P2	0.9964	3.173	0.2184
P3	0.9950	0.6880	1.007
P4	0.8720	0.1081	6.414
P5	0.4934*	0.03220	21.52
P6	0.9998	5.395	0.1285
P7	0.9975	1.050	0.6604
P8	0.9997	2.333	0.2971
P9	NC	NC	NC
P10	0.9949	2.052	0.3377
P11	0.9529	0.2893	2.396

Table S2. The apparent permeabilities (P_{app}) of the peptides tested in the Ussing chamber model, using rat colonic tissue.

**Peptide**	**Time (min)**	**P_{app} (cm/s)**
Oxytocin	60	0.00
	120	0.00
P4	60	5.7×10^{-5}
	120	2.06×10^{-4}
P7	60	0.00
	120	0.00
P11	60	1.20×10^{-5}
	120	3.52×10^{-5}
